# Effectiveness of Autologous Platelet Concentrates in the Sinus Lift Surgery: Findings from Systematic Reviews and Meta-Analyses

**DOI:** 10.3390/dj12040101

**Published:** 2024-04-10

**Authors:** Roberta Gasparro, Alessandro Espedito Di Lauro, Maria Domenica Campana, Nicola Rosiello, Mauro Mariniello, Gilberto Sammartino, Gaetano Marenzi

**Affiliations:** Department of Neurosciences, Reproductive Sciences and Oral Sciences, Section of Oral Surgery, University of Naples Federico II, 80131 Naples, Italy; roberta.gasparro@unina.it (R.G.); alessandroespedito.dilauro@unina.it (A.E.D.L.); mariadomenica.campana@unina.it (M.D.C.); nicolarosiello94@libero.it (N.R.); mauro.mariniello@gmail.com (M.M.); gaetano.marenzi@unina.it (G.M.)

**Keywords:** sinus floor augmentation, bone regeneration, bone substitute, dental implantation, platelet-rich plasma, platelet-rich fibrin

## Abstract

Maxillary sinus augmentation is one of the most predictable procedures for the rehabilitation of the posterior maxilla. The current overview aimed to summarize the findings provided by systematic reviews (SRs) and meta-analyses on the effectiveness of autologous platelet concentrates (APCs) in sinus lift and to assess the methodological quality of the included SRs. Three electronic databases have been explored. SRs and meta-analyses addressing the effectiveness of APCs in sinus lift technique were included. Clinical, radiographic and histomorphometric findings were considered for APCs as solely grafting materials and APCs in combination with biomaterials. Outcomes were implant survival rate (ISR), implant stability (IS), implant failure (IF), postoperative complications, histomorphometric findings, radiographic bone gain, bone volume and bone density. The methodological quality of the included SRs was assessed using the updated version of “A Measurement Tool to Assess Systematic Review” (AMSTAR-2). Thirty SRs were included. The methodological quality of the included reviews ranged from critically low (3 studies) to high (9 studies). The included SRs showed favorable clinical outcomes, short-term new bone formation and no biological complications when APCs were used both as solely graft material or in combination with other biomaterials. However, no significant additional effects in the long-term period were observed. APCs did not add any further positive effects compared to the physiological healing derived by the natural blood clot. The current overview of SRs highlighted the need for high-quality SRs evaluating the role of APCs in sinus lift though network meta-analyses, in order to identify the most powerful material for sinus lift augmentation. The use of APCs improves the healing of soft tissues and the postoperative quality of life in the short-term period. Thus, its application can be recommended.

## 1. Introduction

Implantology has become a well-established treatment option to rehabilitate totally or partially edentulous jaws. An imperative necessity for an implant placement ensuring long-term stability is a proper osseointegration based on a minimum amount of bone width and height of the recipient site. In this regard, one of the challenges for clinicians is represented by the rehabilitation of the atrophic posterior upper jaw, where the progressive expansion of maxillary sinus over the years and the loss of posterior teeth reduces the available bone for a standard implant-prosthetic rehabilitation [1,2,3]. Lateral and transcrestal sinus floor elevation represent the most widely techniques to increase alveolar bone height by the formation of new bone in the maxillary posterior region. A controversial still open issue concerns the material used to fill the newly formed cavity following sinus lift. Numerous biomaterials and bone substitutes have been proposed for application in the maxillary sinus floor lift procedures, mainly to sustain the lifted space. Those include (yet not limited to) autogenous/autograft, freeze-dried bone allograft, xenograft, and alloplastic bone with different successful results [4,5,6,7]. Other authors, instead, have highlighted the considerable regenerative potential deriving from the blood clot alone, not recommending the addition of other grafting material; in these cases, Schneiderian membrane is supported only by the implant apex [8]. To stabilize the blood clot and enhance the healing, biological active molecules, such as bone morphogenic proteins (rhBMPs) [9,10,11], autologous platelet concentrates (APCs) [11,12] and mesenchymal stem cells (MSCs) [13], have been recently introduced as additional or replacement materials in bone augmentation procedures. APCs are biological products derived from the patient’s centrifuged venous blood [14,15,16,17]. Through different methods of centrifugation, it is possible to obtain white blood cells and especially platelets, in a higher quantity than the basal level in peripheral blood. They represent important sources of growth factors and cytokines able to accelerate healing and regeneration of tissues through modulating tissue inflammation, promoting local hemostasis, vascularization of tissues, accelerating new bone formation and improving scaffold mechanics [18]. These biological properties have allowed its use in oral and maxillofacial surgery [19,20,21], dermatology [22], ear–nose–throat surgery [23], plastic surgery [24,25,26], orthopedics [27], sports medicine [28], gynecology [29], cardiovascular surgery [30] and ophthalmology [31]. Systematic reviews (SRs) are considered the best type of publication for gathering existing evidence and providing clinicians with a summary of the latest findings on a clinical question. However, several SRs have been conducted to evaluate the impact of APC in the sinus lift surgery with or without other grafting materials leading to conflicting results due to varying inclusion/exclusion criteria and the quality of primary studies [32,33,34]. To address these difficulties in evaluating evidence and making decisions, the next step is to conduct overviews of SRs. These overviews provide a comprehensive summary of the results from multiple SRs and meta-analyses in an easily understandable format, and they assess the quality of existing SRs on a topic. They are also valuable tools for clinicians to make treatment plans based on the highest level of evidence and for researchers to identify priorities for future research. To the best of our knowledge, this is the first overview conducted on this topic. Therefore, the aim of this overview was to summarize the results from systematic reviews and meta-analyzes regarding the efficacy of the different autologous platelet concentrates, as solely filling material or in association with other biomaterials in the sinus lift surgery and to assess the methodological quality of the included systematic reviews.

## 2. Materials and Methods

This review was compiled following PRISMA (Preferred Reporting Items for Systematic Reviews and Meta-Analyses) guidelines for improving the reporting of systematic reviews and meta-analyses. According to the PICO (P: population, I: intervention, C: comparison, O: outcome) protocol, this overview aimed to answer the following question: “Does the use of autologous platelet concentrates (APCs) as solely grafting material or in association with other biomaterials (Intervention) improve clinical, radiographic and hystomorphometric outcomes (Outcome), in patients undergoing sinus lift surgery, with both crestal and lateral access (Population)?” All APCs described in the current scientific literature were considered “Interventions”, while spontaneous healing of the intervention site associated with the only regenerative power of the blood clot or addition of other different biomaterials were considered as “Comparisons”. Postoperative discomfort and patient-centered outcomes, such as quality of life problems (functional limitations in chewing, speaking, sleeping and inability to perform daily routines and work activities correctly), were considered as secondary outcomes. The protocol was registered on the PROSPERO National Institute of Health Research Database (CRD42023391448).

### 2.1. Literature Search and Review Selection

Initially, a pilot search was conducted on PubMed to check for the presence of existing overviews and collate enough systematic reviews (SRs) that could serve as a solid foundation for the creation of the above-mentioned overview. Literature research was conducted for reviews and meta-analysis published up to 31 March 2023, using three electronic databases (PubMed, Scopus, The Cochrane Library). Different combinations of keywords and MeSH terms, according to the database’s rules, were developed to identify suitable studies. The search strategy is reported in Table 1. A manual search was performed in oral surgery journals (Clinical Implant Dentistry and Related Research, Clinical Oral Implants Research, Journal of Prosthodontic Research Journal of Cranio-Maxillo-Facial Surgery, International Journal of Oral and Maxillofacial Surgery, International Journal of Oral and Maxillofacial Implants, International Journal of Oral Implantology, Journal of Osseointegration) and a further search was performed among the references of the included articles. The grey literature was explored by searching among the conference abstracts published on Web of Science and Scopus and on the databases of scientific dental congresses (International Congress of Oral Implantologists (ICOI), International Association for Dental Research (IADR), European Federation of Periodontology (EFP)). Review selection was performed by two independent reviewers (MDC, RG). Eligibility criteria were SRs and meta-analyses addressing the effectiveness of APCs, as solely grafting materials or in association with other biomaterials, in crestal and lateral sinus lift surgery, in the English language, published up to 31 March 2023. Exclusion criteria were the following: clinical controlled trials (CCTs) and randomized controlled trials (RCTs), dual publications, narrative reviews, case series, questionnaires, radiographic studies, studies with histological data only, animal studies, case reports, letters to the editor, and in vitro studies. Also, abstract and articles written in any language other than English were excluded. After title and abstract screening, the articles were selected for full-text eligibility. Whenever differences in the judgement of the eligibility of title and abstract occurred, full texts were included for final assessment. Disagreements between the two authors were solved by the intervention of a third reviewer (GS).

### 2.2. Data Extraction

Data were independently extracted by two authors (MDC, RG) using a predetermined extraction form. Whenever the information provided in the SRs was not clear, the individual studies were consulted. The authors were not contacted for further details. The following characteristics of each study were extracted: author, publication year, search period, databases, study design (SR with or without meta-analysis), total number of subjects included, intervention and control groups, outcome measures, methods of measurement, quality tool and quality of the individual studies, and the author’s conclusion.

### 2.3. Methodological Quality of Included Reviews

The methodological quality of the included SRs was independently assessed by two reviewers [MDC, NR] using the revised and updated version of A Measurement Tool to Assess Systematic Review (AMSTAR-2). AMSTAR-2 is a valid and reliable instrument made of 16 items, which correspond to three possible responses: “yes”, “partial yes” or “no.” After interpreting the weaknesses detected in critical and non-critical items, the overall quality rating of a SR was reported as “high”, “moderate”, “low” or “critically low”.

## 3. Results

### 3.1. Search Results

**Figure 1:**
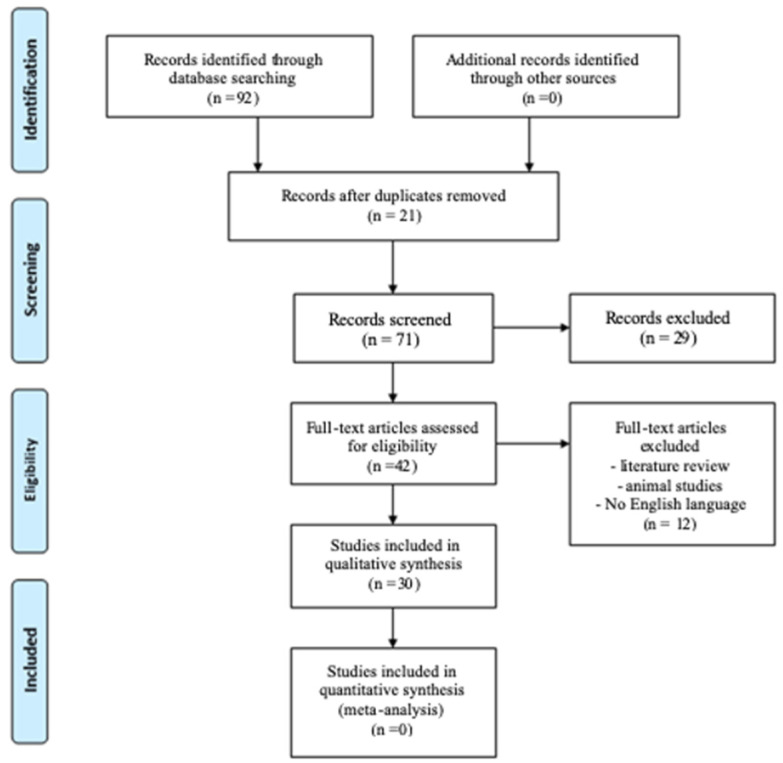
PRISMA flow diagram of the included and excluded records.

Figure 1 shows the flow diagram of study selection. A total of 92 records were identified through electronic and manual search. After duplicates removal, the title and abstracts of 71 records were screened. Of these, 42 manuscripts were included for full-text reading, while 29 were excluded according to the application of the exclusion criteria. For the references of the excluded full-text and reasons, consult the Supplementary Table S1.

Finally, 30 SRs were included for the qualitative analysis [7,32,33,34,35,36,37,38,39,40,41,42,43,44,45,46,47,48,49,50,51,52,53,54,55,56,57,58,59,60].

### 3.2. Characteristics of Included Reviews

Data extracted from the thirty (30) SRs are summarized in Table 2. The number of primary studies included in each SR ranged between two (2) and forty-two (42). Fourteen (14) SRs were integrated with a meta-analysis [34,35,36,39,40,46,47,50,51,54,55,56,60]. Most of the SRs included as primary study clinical controlled studies (CCTs) and randomized clinical trials (RCTs), but eight (8) SRs also included non-controlled studies, case series and case reports [32,34,38,49,52,53,56,59]. None of the included reviews were based on non-controlled studies only; but, in two articles [33,42], the type of included clinical studies is not specified. The number of total subjects included in each review was not always clarified. The initial diagnosis was not clearly reported in some of the included reviews. The surgical procedures studied were lateral and crestal sinus augmentation. APCs were compared to other biomaterials or to the healing provided by blood clot alone or there was no control group. The primary outcomes in most of the studies were clinical (implant success and implant survival), radiographical (bone volume, bone height, bone density) and histomorphometric (percentage of new bone formation). Other reported outcomes were soft-tissue healing, postoperative complications and patient-centered outcomes.

### 3.3. Methodological Quality of Included Reviews

The methodological quality of the included reviews as measured with the AMSTAR-2 ranged from critically low (3 studies) to high (9 studies). The most common critical weakness in the included reviews was the absence of clearly a prior established review methods and any significant deviations from the protocol (Table 3).

**Table 2:** Study Characteristics.

Author, Year of Publication	Search Period	Databases	Study Design; Total No. of Subjects	Diagnosis	Intervention	Control	Quality Tool and Quality of the Individual Studies	Outcome	Conclusion
**Abdalla RIB et al., 2018 [35]**	Up to 6 September 2017	PubMed, Cochrane Library	SR and MA of 4 RCTs; 106 subjects	Subjects with atrophic posterior maxilla	Type of sinus lift not reported. PRP in combination with AB, ABB, DBBM	Biomaterials alone	3 RCTs showed an unclear risk of bias, 1 RCT low risk of bias	Implant failure, complications at treated sites (sinusitis, infection, hemorrhage)	The metanalysis revealed no statistically significant difference between the PRP versus non-PRP groups regarding implant failure and complication rate.
**Ali S et al., 2015 [33]**	From 2006 to 2013	PubMed	SR of 8 clinical studies; 164 subjects	Subjects with atrophic posterior maxilla	Lateral sinus lift using PRF alone or in combination with DFDBA or bovine xenograft	No control group or DFDBA alone or bovine xenograft alone	NR	Implant survival, radiographic bone height, volume and density, hystomorphometric analyses	PRF showed optimistic results as a sole filling material for sinus lift with simultaneous implant placement. Then, it seemed to accelerate maturation of a DFDBA but it had no effect on deproteinized bovine maturation. PRF membranes represent an easy and successful method to cover the sinus membrane or osteotomy window.
**Anitua E. et al., 2022 [36]**	Up to 16 September 2021	PubMed, Cochrane Library, OVID	SR and MA of 3 RCTs and 3 CCTs; 139 subjects	Subjects with atrophic posterior maxilla	Type of sinus lift not reported. P-PRP/L-PRP in combination with ABB	ABB alone	3 RCTs showed a low risk of bias, 2 CCTs moderate, 1 CCT low	Percentage of NBF	A beneficial effect on bone formation after maxillary sinus floor elevation can be obtained when anorganic bovine bone is mixed with PRGF.
**Arora NS et al., 2010 [37]**	From 1950 to 2008	PubMed, Cochrane Library	SR of 5 RCTs; 89 subjects	Subjects with atrophic posterior maxilla	Type of sinus lift not reported. PRP in combination with AB, FDBA, β-TCP	Biomaterials alone	NR	Histological and radiographic evaluation of NBF, early implant placement, quality of life, adverse effects	Although no additional benefit was found in one study, in the others test groups, PRP gave greater bone formation, acceleration of bone formation, higher implant survival rate. Moreover, the handling of the particulate bone grafts was improved.
**Avila-Ortiz G et al., 2016 [38]**	Up to 17 March 2014	PubMed, Web of Knowledge, Scopus, EMBASE, Cochrane Library, ProQuest	RS of 89 studies of which 33 are on PRP/PRGF/PRF in sinus lift: 12 RCTs, 3 non-RCTs, 14 case series, 4 case reports; 754 subjects	Subjects with atrophic posterior maxillary ridge	Lateral and crestal sinus lift using PRP/PRGF/PRF alone or in combination with AB, bovine xenograft, allograft, β-TCP, algae-derived HA, aragonitic calcium carbonate	Biomaterials alone	RCTs showed a level of evidence 2, non-RCTs level of evidence 3, case series and case report level of evidence 4 (Oxford Scale)	Implant survival and success rate, complications, density of the grafted volume, bone height gain, MBL, BIC, histomorphometric measures	The use of blood-derived products did not suppose a significant benefit compared with the diverse control therapies for all the parameters analyzed with the exception of improved short-term bone formation and increased radiographic density.
**Bae JH et al., 2011 [39]**	From 2000 to January 2010	PubMed, Cochrane Library, EMBASE	SR and MA of 6 RCTs and 2 CCTs; 191 subjects	Subjects with atrophic posterior maxillary ridge	Type of sinus lift not reported. PRP in combination with FDBA, AB, ABB	Biomaterials alone	No definite publication bias was found in MA of four studies	Implant survival, percentage of bone formation, BIC	Implant survival and BIC was not significantly different in the intervention group treated with PRP compared to control group; bone formation was significantly greater in PRP group.
**J.V.D.S. Canellas et al., 2021 [40]**	Up to 13 July 2020	PubMed, EMBASE, Cochrane Library, Scopus, Web of Science, LILACS	SR and MA of 11 RCTs of which 2 are on L-PRF;23 subjects	Patients with bone height < 5 mm	Lateral sinus lift using L-PRF in combination with bovine xenograft	Bovine xenograft alone	All studies showed an unclear risk of bias	Percentage of NBF, percentage of residual bone substitute	L-PRF, did not improve bone healing in maxillary sinus floor elevation surgery filled with Bio-Oss.
**Castro AB et al., 2017 [41]**	Up to 31 July 2015	PubMed, EMBASE, Cochrane Library	RS of 14 RCTs of which 3 are on L-PRF in sinus lift; 76 subjects	Subjects with atrophic posterior maxillary	Lateral and crestal sinus lift using L-PRF in combination with xenograft	Xenograft alone	All articles on sinus lift showed a moderate risk of bias	Time and percentage of NBF	When L-PRF was added to xenograft during lateral and crestal sinus floor elevation NBF occurred faster, although the percentage of NBF was not statistically different between test and control groups.
**Damsaz M et al., 2020 [42]**	From January 2009 to 3 February 2020	PubMed, Google Scholar, Cochrane Library	SR of 7 clinical studies of which 6 are on L-PRF in sinus lift; 81 subjects	Subjects with atrophic posterior maxilla	Lateral sinus lift using L-PRF alone or in combination with DBBM or allogenous bone graft	No filling or biomaterials alone	6 studies showed a moderate risk of bias, 1 study high risk of bias	Bone height, time and percentage of NBF, postoperative healing, soft-tissue healing	The addition of L-PRF accelerated bone healing and the amount of regenerated bone but the difference was not statistically different. Postoperative healing was uneventful, without reaching significance.
**Del Fabbro M et al., 2011 [43]**	Up to April 2010	PubMed, EMBASE, Cochrane Library	SR of 12 studies: 10 RCTs, 2 CCT. 269 subjects	Patients with residual ridge height before surgery varied between 1 and 7 mm	Lateral sinus lift using PRP/PRF/PRGF in combination with FDBA, ABB, AB, β-TCP	Biomaterials alone	NR	Implant survival rate, histologic and histomorphometric analysis	No evident benefit can be evidenced regarding clinical outcomes for implant survival. The analysis of hystomorphometric data suggested a possible advantage of using platelet-derived growth factors in new bone formation.
**Dragonas P et al., 2018 [44]**	Up to 20 December 2017	PubMed, Scopus, EMBASE, Cochrane Library, Web of Science, ProQuest, Google Scholar	RS of 17 studies of which 8 are on L-PRF in sinus lift: 6 RCTs and 2 CCTs;NR	Subjects with atrophic posterior maxilla	Lateral and crestal sinus lift using L-PRF in combination with xenograft, FDBA, β-TCP	Biomaterials alone	5 studies showed an high risk of bias, 3 unclear risk of bias	Implant survival, percentage of NBF and bone to bone substitute contact	The use of L-PRF in maxillary sinus augmentation procedures was not associated with more favorable outcomes.
**Dragonas P et al., 2019 [45]**	Up to 23 April 2018	PubMed, Scopus, EMBASE, Cochrane Library, Web of Science, ProQuest and Google Scholar	SR of 8 studies of which 5 are on PRGF in sinus lift: 4 RCTs, 1 CCTs; 158 subjects	Subjects with atrophic posterior maxillary	Lateral sinus lift using PRGF in combination with bovine xenograft or β-TCP	Biomaterials alone	3 studies showed an unclear risk of bias, 2 studies low risk of bias	Percentage of NBF, postoperative complications	The addition of PRGF to sinus augmentation was not beneficial on new bone formation and regeneration. Limitations in daily functions were fewer for the PRGF versus control group during the initial postoperative period.
**Esposito M et al., 2010 [46]**	Up to 7 January 2010	PubMed, Cochrane Library, EMBASE	SR of 10 RCTs of which 4 are on PRP; 114 subjects MA of 3 RCTs on PRP in sinus lift	Subjects with atrophic posterior maxillary	Lateral sinus lift using PRP in combination with AB or bovine xenograft	Biomaterials alone	3 studies showed an high risk of bias, 1 low risk of bias	Prothesis failure, bone gain (mm or percentage), major complication at bone donor site, duration of the treatment period	No clinical benefit could be observed in any of the trials when using PRP; therefore, there appear to be no reasons to justify its use in this application.
**Esposito M et al., 2014 [47]**	Up to 17 January 2014	PubMed, Cochrane Library, EMBASE	RS and MA of 18 RCTs on sinus lift of which 2 are on PRP; 62 subjects	Subjects with atrophic posterior maxillary	Lateral sinus lift using PRP with AB or bovine xenograft	Biomaterials alone	1 RCT showed an unclear risk of bias, 1 RCT an high risk of bias	Prosthetic, implant and graft failures, complications, and histomorphometric evaluation	There were no statistically significant differences between groups who received PRP and those who did not for implant failures and complications.
**Fujioka-Kobayashi M. et al., 2021 [48]**	Up to June 2020	PubMed, Cochrane Library, Scopus Embase, LILACS	RS of 18 studies of which 6 RCTs and 4 CCTs are on sinus lift; NR	Subjects with atrophic posterior maxillary	Lateral sinus lift using PRF alone or in combination with AB, DBBM, β-TCP	No control group or biomaterials alone	8 studies showed a low risk of bias, 2 studies unclear risk	NFB, residual bone graft, implant survival rate, postoperative complications	No significant improvement was found in NFB when PRF was added to biomaterials. Only two articles showed an accelerated healing.
**Ghanaati S et al., 2018 [49]**	Up to May 2017	PubMed	RS of 72 studies of which 8 are on PRF in sinus lift: 6 prospective CCTs studies, 1 is quasi-experimental study, 1 is case control study; 198 subjects	Severe maxillary bone atrophy (in 2 studies bone height < 5 mm)	Lateral and crestal sinus lift using PRF/L-PRF alone or in combination with AB, FDBA, DBBM	No control group or biomaterials alone	6 studies are IIa, 1 is IIb, 1 is III level of scientific evidence according to US Agency for Healthcare Research and Quality	NFB, bone gain, implant survival rate and implant failure, bone height gain and resorption, periimplant bone density, postoperative complications	No statistically significant differences were found in the addition of PRF to biomaterial in sinus lift compared to biomaterials alone.
**Guo T et al., 2020 [34]**	Up to April 2019	PubMed, Scopus, Cochrane Library	SR and MA of 8 retrospective cohort studies, 6 prospective cohort studies, 2 RCTs, 1 CCTs, 1 not clearly defined; NR	Subjects with atrophic posterior maxilla	Transcrestal sinus floor elevation using PRP, PRF, CGF, PRGF	Blood clot alone	1 trial low risk of bias, 2 trials moderate risk of bias, 15 trials high quality with low risk of bias	Implant survival rate, MBL, endo-sinus bone gain	No significant differences were observed 1-year postsurgery on implant survival rate, MBL, and endosinus bone gain. Then, grafting platelet concentrations around dental implants at transcrestal sinus floor elevation sites did not significantly enhance the adjacent bone regeneration.
**Lemos CA et al., 2015 [32]**	From January 2000 to 20 January 2015	PubMed, EMBASE, Cochrane Library	SR of 12 RCTs and 5 prospective studies; 369 subjects Meta-analysis of 13 RCTs	Subjects with atrophic posterior maxillary	Type of sinus lift not reported. PRP in combination with AB, FDBA, bovine xenograft, β-TCP, algae-derived HA	Biomaterials alone	12 studies showed an high quality, 5 studies low quality	Percentage of NBF, implant survival rate, ISQ, BIC, MBL and alveolar bone height	No influence of PRP with bone graft on NBF, implant survival and stability, MBL and alveolar bone height in maxillary sinus augmentation.PRP can used to facilitate the handling of bone grafts when they are particulate.
**Liu R et al., 2019 [50]**	NR	PubMed, EMBASE, Cochrane Library	MA of 5 RCTs; 133 subjects	Subjects with atrophic posterior maxillary	Type of sinus lift not reported. PRF/PRP in combination with allogenous, xenograft and β-TCP	Biomaterials alone	All studies showed an high risk of bias	Implant survival rate, complications, histological and histomorphometric evaluation (percentage of NBF, residual bone graft, contact between newly formed bone substitute and bone, soft-tissue area)	There were no statistical differences in survival rate, complications, new bone formation, contact between newly formed bone and bone substitute, percentage of residual bone graft and soft-tissue area between the non-PRF and PRF groups.
**Meng Y et al., 2020 [51]**	Up to 31 December 2019	PubMed, Web of Science, EMBASE, Cochrane Library	SR and MA of 11 RCTs; 141 subjects	Subjects with atrophic posterior maxillary	Type of sinus lift not reported. PRF/PRP in combination with AB, DBBM, FDBA, β-TCP	Biomaterials alone	5 studies showed a moderate risk of bias, 6 studies high risk of bias	Percentage of NFB, percentage of residual bone substitute material, percentage of soft-tissue area, radiographic bone density	PRF and PRP did not show additional effect on new bone formation and implant stability when combined with osteoconductive materials. The percentage of residual bone substitute material was not significant between APC group and non-APC group.
**Ortega-Mejia H et al., 2020 [52]**	Up to 9 December 2019	PubMed, Cochrane Library	RS of 23 studies: 9 RCTs, 1 CCT, 2 case series, 5 retro-spective studies, 5 prospective studies; 547 subjects	Subjects with atrophic posterior maxillary	Type of sinus lift not reported. PRF, PRGF, i-PRF alone or PRF in combination with AB, DFDBA, DBBMA, synthetic nanocrystalline HA, β-TCP	Blood clot or biomaterials alone	Reported for only 9 RCTs: 7 studies showed an high risk of bias, 1 study unclear, 1 study low	Percentage of NBF, bone height, implant stability and implant survival, postoperative complications	No additional beneficial effects in terms of augmented bone height, implant survival rate and implant stability. The percentage of NBF was slightly higher in the PRF group, but this was not statistically significant.
**Otero AIP et al., 2022 [53]**	From January 2006 to August 2020	PubMed, Science Direct, Scopus	SR of 6 RCTs, 5 CCTs, 2 retro-spective CTs, 1 clinical-histologic study, 1 case report; 354 subjects	Subjects with atrophic posterior maxillary	Lateral and crestal sinus lift using PRF alone or in combination with bovine xenograft, β-TCP, cortico-cancellous bone, FDBA	PRF alone or with allograft, bovine xenograft, synthetic bone graft or biomaterials alone	8 CTs showed an high quality, 1 CT medium quality, 3 RCTs medium risk of bias, 3 RCTs high risk of bias	Clinical outcomes, bone gain and density	The application of PRF, either alone or with another biomaterials, has been suggested as an effective biomaterial reducing the time for new bone formation. No significant difference was found between groups in terms of ISQ.
**Pocaterra A et al., 2016 [54]**	Up to 3 November 2014	PubMed, Cochrane Library, CINAHL, Science Direct, ISI Web of Knowledge, Scopus	SR of 7 RCTs; MA of 6 RCTs; 155 subjects	Subjects with atrophic posterior maxillary	Type of sinus lift not reported. PRP in combination with AB, FDBA, ABB	Biomaterials alone	All studies showed an high risk of bias	BIC, percentage of NBF, implant survival	The results of the MA seem to indicate that PRP does not provide additional benefits in newly bone formation or improve the implant survival rate.
**Rickert D et al., 2011 [55]**	Up to September 2010	PubMed, EMBASE	SR of 12 RCTs of which 4 are on PRP in sinus lift; 73 subjects. MA of 5 RCTs of which 2 are on PRP; 23 subjects	Subjects with atrophic posterior maxillary	Type of sinus lift not reported. PRP in combination with AB	AB alone	NR	Percentage of NBF, implant survival	Adding PRP to grafting material did not promote new bone formation and implant survival.
**Schliephake H, 2013 [56]**	From 1995 to 2012	PubMed, Cochrane Library	SR of 42 studies: 3 case reports, 9 case series, 5 cohort studies (n° subjects NR); 6 RCTs, 14 cohort studies (373 sub.); 5 SRs e MA	Subjects with atrophic posterior maxilla	Type of sinus lift not reported. PRP/PRF alone or in combination with AB, allograft and bovine xenograft	No control group or biomaterials alone	NR	Percentage of NBF, bone density, bone implant contact, perimplant bone height, implant stability, implant survival	No benefit for the final outcome could be shown for the use of PCs in sinus lift procedures.
**Sivakumar I. et al., 2023 [57]**	Up to April 2021	PubMed, Cochrane Library, Scopus	6 RCTs; 188 subjects	Subjects with atrophic posterior maxillary	Type of sinus lift not reported. PRP alone	Biomaterials alone	3 studies showed a low risk of bias, 3 studies unclear risk of bias	Cumulative survival and success of dental implants	The effect of platelet-rich plasma is uncertain on the survival of dental implants.
**Stähli A et al., 2018 [58]**	Up to 31 December 2017	PubMed, EMBASE, Cochrane Library	RS of 22 studies of which 12 are on PRP in sinus lift: 7 RCTs, 5 CCTs; 374 subjects	Subjects with atrophic posterior maxillary	Type of sinus lift not reported. PRP in combination with AB, BBG, DBBM, β-TCP	Biomaterials alone	9 studies showed a moderate risk of bias, 3 studies high risk of bias	Alveolar bone regeneration, soft-tissue healing, graft resorption, osseointegration, postoperative life quality	PRP/PRGF combined with grafting materials may transiently enhance bone formation and reduce postoperative pain and swelling.
**Stumbas A et al., 2019 [59]**	From 1 January 2008 to 1 January 2019	PubMed	RS of 18 retrospective and prospective studies, clinical trials, case–control and case series studies. Of these articles, 4 are on PRP/L-PRF in sinus lift	Subjects with atrophic posterior maxillary	Lateral sinus lift using PRP/PRF in combination with AB and bovine xenograft	Biomaterials alone	All articles showed an unclear risk of bias	Percentage of NBF, residual graft particles, and soft-tissue healing	PRP combined together with bone graft materials enhances bone formation and vascularization; it might also reduce inflammation and the risk of complications.
**Suárez-López del Amo F. et Monje A., 2022 [60]**	From January 2000 to October 2021	PubMed, Cochrane Library, EMBASE	SR of 12 RCTs of which 7 are on APCs; 100 subjects	Subjects with atrophic posterior maxillary	Lateral and crestal sinus lift using APCs alone or in combination with AB, DBBM, TCP, CaP	No control group or biomaterials alone	92% of the studies present some concerns, while 8% of studies show low risk of bias	Data on linear and volumetric dimensional changes by CT, percentage of NBF	In mostly studies APCs do not improve linear and volumetric dimensional changes and the amount of new bone formation.
**Trimmel B. et al., 2021 [7]**	Up to 1 October 2019	PubMed, Cochrane Library, EMBASE, EBSCO, WOS	SR and MA of 34 RCTs of which 9 are on APCs in sinus lift; NR	Subjects with atrophic posterior maxillary	Lateral sinus lift using PRF/PRP/PRGF in combination with AB, bovine xenograft, β-TCP, nanocrystalline HA	Biomaterials alone	7 studies showed an unclear risk of bias, 2 high risk of bias	Percentage of NBF	The combination of biomaterials with APCs represents a feasible alternative for AB substitution to achieve high NBF levels with the conventionally used 5- to 8-month healing periods.

SR, systematic review; MA, meta-analysis; RCTs, randomized control trials; PRP, platelet-rich plasma; AB, autogenous bone; ABB, anorganic bovine bone; DBBM, deproteinized bovine bone mineral; PRF, platelet-rich fibrin DFDBA, demineralized freeze-dried bone allograft; CCTs, clinical controlled trials; P-PRP, pure-platelet-rich plasma; L-PRP, leukocyte-platelet-rich fibrin; PRGF, platelet-rich growth factors; NBF, new bone formation; β-TCP, β-tricalcium phosphate; HA, hydroxyapatite; MBL, marginal bone loss; BIC, bone to implant contact; L-PRF, leukocyte, platelet-rich fibrin; CGF, concentrates growth factor; ISQ, implant stability quotient; BBG, bovine bone graft; CaP, calcium phosphate.

### 3.4. Clinical, Radiographical and Histomorphometric Results

To make the reading of the results simpler, we divided them into two specific categories: APCs as solely grafting material and APCs in combination with other biomaterials.

#### 3.4.1. APCs as Solely Grafting Material

There is only one SR [34] that discusses the effects of APCs alone vs. blood clot. Eight SRs include studies that examined APCs alone and APCs in association with other biomaterials as control [33,38,42,49,52,53,56,60].

Guo T. et al. [34] reported no significant differences between the 1-year implant survival rate of the non-grafted group (97%) and the APCs group (99%). Moreover, Ali and coworkers [33] reported high implant survival rates although only one primary study [61,62] provided a long-term follow-up (2–6 years). No postoperative complications were observed during the healing period. In the few cases in which a sinus membrane’s perforation occurred, it was solved by PRF membrane thanks to its good intrinsic adherence to the Schneiderian membrane [33,53].

In relation to radiographic bone height, volume and density and marginal bone loss (MBL), most of the included SRs agreed that APCs were a reliable method that could lead to short-term new bone formation but without long-term significant differences [38,42,53,55].

According to Guo T. [34], there was postsurgical endo-sinus bone gain with the highest value of 8.23 + 2.88 mm at 14 months postsurgery. Similarly, other SRs [38,42,49,52] reported the highest level of vertical bone gain between 8.5 and 12 mm, using L-PRF as sole filling material.

Ali [33] showed values of 0.7 mL + 0.31 mL of bone volume and 323 + 156.2 Hounsfield Units (HU) of bone density. On the contrary, Ortega-Meja [52] pointed out that the allograft group had a statistically significant superior bone volume gain (53%), bone density (86%) and height (69%) compared to Titanium-PRF.

In regard to histological and histomorphometric evaluation, instead, Ali’s and Ortega-Meja’s reviews [33,52] showed that PRF was able to create a bone matrix more than 30% after six months of follow-up.

#### 3.4.2. APCs in Combination with Other Biomaterials

Twenty-seven reviews include articles regarding the use of APCs in combination with other grafting materials [7,30,31,32,33,34,35,36,37,38,39,40,41,42,43,44,45,46,49,50,51,52,53,54,55,56,57,58]. Among these, eight SRs [33,38,42,49,52,53,56,60] also included articles regarding APCs alone vs. blood clot while twenty-one SRs only included articles regarding APCs in combination with other biomaterials and biomaterials alone as control [7,32,35,36,37,39,40,41,43,44,45,46,47,48,50,51,54,55,57,58,59]. No significant differences regarding implant failure and complications (e.g., sinusitis, infection, hemorrhage) were found between the APCs/biomaterials and APCs alone [32,35,47,55,57].

Similar findings were observed by Dragonas et al. [44], reporting no statistically significant difference in the implant stability quotient (ISQ) at any of the follow-up periods (106, 120, 150 days) [63]. About quality of life, gradual improvements in postoperative pain, swelling, sleeping, eating, phonetics, activities of daily living and number of missed working days in the L-PRF group were reported over the first 7 postoperative days; however, no significant differences between the two groups were observed [42,44].

Regarding soft-tissue healing, the studies in which APCs were also used as a membrane [44] reported superior outcomes in terms of tissue color, response to palpation, presence/absence of granulation tissue, and incision margin opening but the differences were not statistically significant versus the control group.

About bone gain, Otero et al. [53] reported that the application of PRF either alone or in conjunction with another biomaterial is an effective biomaterial, reducing the time for new bone formation and, consequently, the time necessary for implant rehabilitation. Two SRs [37,39] showed a statistically higher bone volume and bone density in the short-term period (6 months/1-year) between the test and control groups but, in the long term, no statistically significant differences were found. Also, when PRF was mixed with deproteinized bovine bone, the authors reported a 31% increase in the perimplant bone density [49]. Nevertheless, Fujioka-Kobayashi M. et al. did not report any significant improvement in bone gain when PRF was added to biomaterials [48].

Regarding histological and histomorphometric evaluation, in lateral sinus lift, NBF in the L-PRF group was 1.4-fold higher than that in the control group (deproteinized bovine bone alone) but without statistical significance [41,42]. Therefore, no differences were found in the bone grafts remnants, fibrous tissue within the sinus and percentage of the bone graft in contact with the newly formed bone.

With similar results, pooled analysis of Ortega-Meja [52] showed a slightly higher percentage of NBF in the PRF group when compared to graft materials alone. Anitua et al. [36] discussed that most of the included studies indicated a higher new bone formation in the P-PRP group while only one study showed no differences [64].

Although L-PRF and PRGF did not show a greater proportion of vital bone formation and residual grafting material [33,44,45], they seemed to accelerate bone maturation, reduce the amount of biomaterial needed and reduce the healing time.

Moreover, several SRs [37,39,43,55] demonstrated a significant increase in vital bone regeneration (over 22 + 9% of bone formation) and a decrease in residual graft particles in test sites with a slow resorption of biomaterials and with the capacity of APCs to enhance the osteoconductive nature of freeze-dried bone allograft. Meanwhile, Otero et al. [53] mentioned that the histomorphometric examination of FDBA/PRF revealed 65% of vital new bone and 35% of inert bone within the trabecular areas after 4 months versus, respectively, 69% and 31% in the control group after eight months.

In addition, Stahli et al. [58] reported that PRP can improve the regenerative potential of anorganic bovine bone by increasing the newly formed bone volume (31%) thanks to the stimulation of the vascularization process through the release of growth factors. Finally, the percentage of soft-tissue area was higher in the PRF group (3.73%, 95% CI. 10.11 to 2.66; *p* = 0.25) but no significant difference was detected [32,51].

## 4. Discussion

Rehabilitation of the atrophic posterior maxilla is a challenge for clinicians. Maxillary sinus floor elevation is considered a reliable standardized procedure to achieve a more suitable condition for implant placement in terms of bone height and volume. Many bone substitutes have been proposed to fill the subsinusal neocavity. In this overview, emphasis was focused on the use of growth factors directly derived from centrifugation of patients’ blood samples. Due to wide and conflicting conclusions in the scientific literature discussing the use of APCs in sinus lift surgery, the rationale of this article based on the assessment of systematic reviews dealing with this topic. So, an overview of thirty (30) SRs and meta-analyses regarding the effectiveness of APCs in maxillary sinus augmentation surgery was created. Hence, the purpose was to provide surgeons with some clinical indications about the use of platelet concentrates, both as sole grafting material or in combination with other different bone substitutes in lateral and transalveolar sinus lift surgery. Most SRs provided positive results in terms of clinical, radiographic and histomophometric findings when APCs were used as solely grafting material or in combination with biomaterials. Specifically, better results in implant stability and implant survival rate were found in test groups rather than control groups [37]. APCs also seemed to induce an increased radiographic density and accelerate bone formation and maturation of biomaterial used in combination with [33,37,38,41,42,53,58], although in a short period of follow-up. In fact, a follow-up higher than 6 months/1 year showed a no statistically difference between the APCs group and the non-APCs group in terms of implant stability and implant survival rate [32,39,43,44,47,49,50,51,52,53,54,55,56], radiographic bone gain and bone density and percentage of NBF [32,34,41,42,44,45,46,49,50,51,52,54,55,56,60]. The idea to use APCs in that case derived from the potential role of these concentrates to accelerate and promote soft and hard-tissue healing. Indeed, platelets and leucocytes contain high levels of bioactive mediators such as platelet-derived growth factor (PDGF), vascular endothelial growth factor (VEGF), fibroblast growth factor (FGF), transforming growth factor-β1 (TGF-b1), β2 (TGF-b2) and insulin-like growth factor (IGF), which stimulate angiogenesis, cell proliferation and matrix remodeling [65]. Different types of APCs have been introduced and different methods of preparations have been proposed, producing different percentages of platelets, leukocytes, growth factors and fibrin matrix. For example, tetramolecular fibrin network of PRP release more FGF and PRGF than PRF, so it can improve tissue repair stimulating fibroblast cells. Instead, tridimensional fibrin complex of PRF allow a slow release of a greater amount of VEGF and TGF-β up to seven days later. For this reason, its main role is to improve angiogenesis and tissue regeneration. Similarly to PRF, an even greater amount of growth factors, such as VEGF and FGF, are isolated in CGF so it would seem to show superior regenerative capacity, as reported for sinus and alveolar ridge augmentation [66]. In the current overview, there were no restrictions regarding the inclusion of all types of APCs, so the abovementioned variability can influence the macroscopic characteristics of the APCs and biological properties and, consequently, have an impact on the final outcomes.

Another limit can be represented by the fact that, when APCs are used with other biomaterials, the role of APCs may be hidden by the bone graft material. So, the contribution of each of them was not clearly specified. The use of APCs as a membrane also had to be taken into account. In fact, in this form, they represent an easy and successful method to cover the Schneiderian membrane or osteotomy window. So, they might repair an eventual sinus membrane perforation and improve soft-tissue healing beyond bone/hard-tissue healing when using as a filling material [33,41,44]. We also must consider that in some studies [33,35,39,41,42], the type of surgical approach (lateral or crestal) and the time of implant insertion were not always specified. However, we can suppose that, by using APCs alone with a lateral approach, the time of implant placement is contemporary to surgery. In fact, APCs alone could not have supported the raised sinus floor as implants can. Similarly, when authors report histomorphometric analysis, the type of surgery could be clarified because bone biopsies have been obtained during second stage of implant surgery. Furthermore, most of the studies cited in this review do not account for a multitude of data, beyond type of surgical approach and implant placement protocols: patients’ number, specific diagnosis of the sample selected, especially in terms of residual bone height, absence of a control group that made not possible the evaluation of the effectiveness of APCs in sinus elevation [33,49,56,60]. Beyond these considerations, the majority of the clinical trials did not also account for the maxillary sinus width which represents a significant value, with the residual bone height, in the choice between lateral or crestal surgical approach [67]. This parameter can influence the percentage of new bone formation after sinus lift surgery. Indeed, the wider is the sinus, the longer is the path that osteoprogenitor cells derived from sinus walls have to go through. On the contrary, the narrower the sinus, the more bone formation [68,69]. Another limitation is that most of the included studies reported a high or unclear risk of bias, while it was not even explained in other ones. Therefore, the quality of primary studies is highly variable, and the risk of bias is judged as low only in a few cases. Thus, it is critical for clinicians and researchers to assess the reliability of the results derived from these studies. Qualitative analysis of the included SRs was made using the AMSTAR scale, which is a validated tool for the methodological quality assessment of SRs [70]. The current overview showed a range from critically low (3 studies) to high (9 studies) methodologically quality, indicating a moderate overall quality. The main critical weakness was the absence of an explicit statement that the review methods were established prior to the conduct of the review, such as the presence of a registered protocol on PROSPERO (International Prospective Register of Ongoing Systematic Reviews), an online database which informs the whole scientific community about topics covered providing a pre-established review’s method [71]. Justification of any significant deviations from the protocol were also absent. The results of the current overview should be interpreted with caution due to certain limitations. Despite the thorough search process, it is possible that some relevant literature may have been overlooked. Furthermore, most of the existing literature has only reported short-term results with an average follow-up time of 3–6 months.

## 5. Conclusions

The current overview of SRs highlighted that the quality level of the published SRs focusing on the topic of APCs in sinus lift was extremely variable, thus ranging from low to high. According to clinical and histological results, it has demonstrated that the use of APCs seems to be a reliable surgical option promoting natural bone regeneration, providing a superior support for the elevated sinus membrane, and working as a shield against soft-tissue invagination and ingrowth. However, there is limited evidence on the potential benefits of APCs in long-term bone regeneration and soft-tissue healing. In fact, there still appears to be a lack of sufficient scientific support to justify their convenience. Thus, the search for components not only able to maintain the space necessary for bone regeneration but also to stimulate new bone formation continues. Therefore, this overview emphasizes the need to further investigate the role of APCs in the future through studies characterized by a higher sample size, standardization of the preparation protocol of the concentrates, and a longer follow-up.

## Figures and Tables

**Table 1:** The search strategy for each database and relative results.

Databases	Search Strategy
**PubMed**	((“sinus lift” [All Fields]) OR (“sinus lifting” [All Fields]) OR (“sinus augmentation [All Fields]”) OR (“sinus elevation [All Fileds]”) OR (“maxillary sinus lift” [All Fields]) OR (“maxillary sinus elevation” [All Fields]) OR (“maxillary sinus augmentation [All Fields]”) OR (“maxillary sinus floor elevation” [All Fields]) OR (“maxillary sinus floor lift” [All Fields]) OR (“maxillary sinus floor augmentation” [All Fields] OR (“maxillary sinus/surgery” [MeSH Terms]) OR (“sinus floor augmentation” [MesH Terms])) AND ((“prp” [All Fields]) OR (“platelet rich plasma” [All Fields]) OR (“prf” [All Fields]) OR (“platelet rich fibrin” [All Fields]) OR (“autologous platelet concentrates” [All Fields]) OR (“platelet concentrates” [All Fields]) OR (platelet concentrations-grafted [Title/Abstract])) AND ((meta-analysis [Filter] OR systematic review [Filter]))
**Scopus**	(TITLE-ABS-KEY (sinus lift) OR TITLE-ABS-KEY (sinus lifting) OR TITLE-ABS-KEY (sinus augmentation) OR TITLE-ABS-KEY (sinus elevation) OR TITLE-ABS-KEY (maxillary sinus lift) OR TITLE-ABS-KEY (maxillary sinus elevation) OR TITLE-ABS-KEY (maxillary sinus augmentation) OR TITLE-ABS-KEY (maxillary sinus floor elevation) OR TITLE-ABS-KEY (maxillary sinus floor lift) OR TITLE-ABS-KEY (maxillary sinus floor augmentation) OR TITLE-ABS-KEY (maxillary sinus/surgery) OR TITLE-ABS-KEY (sinus floor augmentation)) AND (TITLE-ABS-KEY (prp) OR TITLE-ABS-KEY (platelet rich plasma) OR TITLE-ABS-KEY (prf) OR TITLE-ABS-KEY (platelet rich fibrin) OR TITLE-ABS-KEY (autologous platelet concentrates) OR TITLE-ABS-KEY (platelet concentrates) OR TITLE-ABS-KEY (platelet concentrations-grafted)) AND (LIMIT-TO (DOCTYPE, “re”))
**Cochrane**	(Platelet concentrates in maxillary sinus lift):ti,ab,kw

**Table 3:** Quality assessment of the included systematic reviews, according to the AMSTAR-2.

			Abdalla RIB et al., 2018 [35]	Ali S et al., 2015 [33]	Anitua E. et al., 2022 [36]	Arora NS et al., 2010 [37]	Avila-Ortiz G et al., 2016 [38]	Bae JH et al., 2011 [39]	J.V.D.S. Canellas et al., 2021 [40]	Castro AB et al., 2017 [41]	Damsaz M et al., 2020 [42]	Del Fabbro M et al., 2011 [43]	Dragonas P et al., 2018 [44]	Dragonas P et al., Mar 2019 [45]	Esposito M et al., 2010 [46]	Esposito M et al., 2014 [47]
Did the research questions and inclusion criteria for the review include the components of PICO?			N	N	Y	N	Y	N	Y	Y	Y	N	Y	Y	N	N
Did the report of the review contain an explicit statement that the review methods were established prior to the conduct of the review and did the report justify any significant deviations from the protocol?			N	N	N	N	N	N	N	N	N	N	N	N	N	N
Did the review authors explain their selection of the study designs for inclusion in the review?			Y	PY	Y	Y	Y	Y	Y	Y	PY	Y	Y	Y	Y	Y
Did the review authors use a comprehensive literature search strategy?			Y	N	Y	Y	Y	Y	Y	Y	Y	Y	Y	Y	Y	Y
Did the review authors perform study selection in duplicate?			Y	Y	Y	Y	Y	Y	Y	Y	Y	Y	Y	Y	Y	Y
Did the review authors perform data extraction in duplicate?			Y	Y	N	Y	N	Y	NR	Y	Y	Y	Y	Y	Y	Y
Did the review authors provide a list of excluded studies and justify the exclusions?			Y	N	Y	Y	Y	Y	N	Y	N	N	Y	Y	Y	Y
Did the review authors describe the included studies in adequate detail?			Y	PY	Y	Y	Y	Y	Y	Y	Y	Y	Y	Y	PY	Y
Did the review authors use a satisfactory technique for assessing the risk of bias (RoB) in individual studies that were included in the review?			Y	N	Y	N	Y	N	Y	Y	PY	N	Y	Y	Y	Y
Did the review authors report on the sources of funding for the studies included in the review?			N	N	Y	Y	Y	Y	N	Y	Y	N	Y	Y	N	Y
If meta-analysis was performed did the review authors use appropriate methods for statistical combination of results?			Y	Nm	Y	Nm	Nm	Y	Y	Nm	Nm	Nm	Nm	Nm	Y	Y
If meta-analysis was performed did the review authors assess the potential impact of RoB in individual studies on the results of the meta-analysis or other evidence synthesis?			Y	Nm	Y	Nm	Nm	N	Y	Nm	Nm	Nm	Nm	Nm	Y	Y
Did the review authors account for RoB in individual studies when interpreting/discussing the results of the review?			Y	N	Y	N	Y	N	Y	Y	N	N	Y	N	Y	Y
Did the review authors provide a satisfactory explanation for, and discussion of, any heterogeneity observed in the results of the review?			Y	PY	Y	Y	Y	Y	Y	Y	Y	Y	Y	Y	Y	Y
If they performed quantitative synthesis did the review authors carry out an adequate investigation of publication bias (small study bias) and discuss its likely impact on the results of the review?			N	Nm	N	Nm	Nm	N	Y	Nm	Nm	Nm	Nm	Nm	N	N
Did the review authors report any potential sources of conflict of interest, including any funding they received for conducting the review?			N	N	Y	Y	Y	Y	Y	Y	Y	N	Y	Y	Y	Y
OVERALL QUALITY ASSESSMENT			**M**	**L**	**H**	**M**	**L**	**M**	**H**	**M**	**L**	**L**	**M**	**M**	**M**	**H**
	**Fujioka-Kobayashi M., 2021 [48]**	**Ghanaati S et al., 2018 [49]**	**Guo T et al., 2020 [34]**	**Lemos CA et al., 2015 [32]**	**Liu R et al., 2019 [50]**	**Meng Y et al., 2020 [51]**	**Ortega-Mejia H et al., 2020 [52]**	**AI P Otero et al., 2022 [53]**	**Pocaterra A et al., 2016 [54]**	**Rickert D et al., 2011 [55]**	**Schliephake H, 2013 [56]**	**Sivakumar I. et al., 2023 [57]**	**Stähli A et al., 2018 [58]**	**Stumbas A et al., 2019 [59]**	**Suárez-López del Amo F. et Monje A., 2022 [60]**	**Trimmel B. et al., 2021 [7]**
Did the research questions and inclusion criteria for the review include the components of PICO?	Y	N	Y	Y	N	Y	Y	Y	N	N	N	Y	Y	Y	Y	Y
Did the report of the review contain an explicit statement that the review methods were established prior to the conduct of the review and did the report justify any significant deviations from the protocol?	N	N	N	N	N	N	N	N	N	N	N	N	N	N	N	N
Did the review authors explain their selection of the study designs for inclusion in the review?	Y	Y	Y	Y	Y	Y	Y	Y	Y	Y	Y	Y	Y	Y	Y	PY
Did the review authors use a comprehensive literature search strategy?	Y	N	Y	Y	Y	Y	Y	Y	Y	Y	Y	Y	Y	N	Y	Y
Did the review authors perform study selection in duplicate?	Y	N	Y	Y	Y	Y	Y	Y	Y	Y	NR	Y	Y	NR	Y	Y
Did the review authors perform data extraction in duplicate?	Y	Y	Y	Y	Y	Y	Y	N	Y	Y	NR	Y	Y	Y	Y	Y
Did the review authors provide a list of excluded studies and justify the exclusions?	N	N	Y	Y	N	N	Y	Y	Y	N	N	N	Y	Y	Y	Y
Did the review authors describe the included studies in adequate detail?	Y	Y	Y	Y	Y	Y	Y	Y	Y	Y	N	Y	Y	PY	Y	Y
Did the review authors use a satisfactory technique for assessing the risk of bias (RoB) in individual studies that were included in the review?	Y	N	Y	Y	Y	Y	PY	Y	Y	N	N	Y	Y	Y	Y	Y
Did the review authors report on the sources of funding for the studies included in the review?	N	Y	Y	Y	Y	Y	Y	Y	Y	Y	N	Y	Y	N	Y	Y
If meta-analysis was performed did the review authors use appropriate methods for statistical combination of results?	Nm	Nm	Y	Y	Y	Y	Nm	Nm	Y	Y	Nm	Nm	Nm	Nm	Nm	Y
If meta-analysis was performed did the review authors assess the potential impact of RoB in individual studies on the results of the meta-analysis or other evidence synthesis?	Nm	Nm	N	Y	Y	Y	Nm	Nm	Y	N	Nm	Nm	Nm	Nm	Nm	Y
Did the review authors account for RoB in individual studies when interpreting/discussing the results of the review?	Y	N	Y	Y	Y	Y	Y	Y	Y	N	N	Y	Y	N	Y	Y
Did the review authors provide a satisfactory explanation for, and discussion of, any heterogeneity observed in the results of the review?	Y	Y	Y	Y	Y	Y	Y	Y	Y	Y	N	Y	Y	Y	Y	Y
If they performed quantitative synthesis did the review authors carry out an adequate investigation of publication bias (small study bias) and discuss its likely impact on the results of the review?	Nm	Nm	N	N	N	N	Nm	Nm	Y	N	Nm	Nm	Nm	Nm	Nm	Y
Did the review authors report any potential sources of conflict of interest, including any funding they received for conducting the review?	Y	Y	Y	Y	Y	Y	Y	Y	Y	Y	N	Y	Y	Y	Y	Y
OVERALL QUALITY ASSESSMENT	**M**	**CL**	**H**	**H**	**H**	**H**	**M**	**M**	**H**	**L**	**CL**	**M**	**M**	**CL**	**M**	**H**

Y: Yes; N: No; Nm: No metanalysis; CL: Critically Low; L: Low, M: Medium; H: High.

## Supplementary Materials

The following supporting information can be downloaded at: https://www.mdpi.com/article/10.3390/dj12040101/s1, Table S1: References excluded and reason for the exclusion. References [4,12,72,73,74,75,76,77,78,79,80,81] are cited in the supplementary materials.
