# Supplementary material for: Effectiveness of Autologous Platelet Concentrates in the Sinus Lift Surgery: Findings from Systematic Reviews and Meta-Analyses
Source: Dent J (Basel). 2024 Apr 10;12(4):101. doi: 10.3390/dj12040101 (PMC11049363; doi:10.3390/dj12040101)
Supplement: Supplementary file 1 [file dentistry-12-00101-s001.zip › dentistry-2877445-supplementary.pdf]

Table S1: References excluded and reason for the exclusion.

Reference	Exclusion criteria
Al-Hamed FS et al, 2019 [72]	Narrative Review
Al-Moraissi EA et al, 2020 [12]	Main topic is related to bone substitutes, not on APCs
Bernardi S et al, 2020 [73]	Narrative Review
Dai YZ et al, 2011 [74]	Narrative Review
Esposito M et al, 2006 [75]	Inability to summarize the data
Farshidfar N et al, 2022 [76]	Narrative Review
Franchini M et al, 2019 [77]	Lack of conclusion on the efficacy of PRF in sinus lift
Kumar KR et al, 2016 [78]	Narrative Review
Maniyar N, 2018 [79]	Narrative Review
Miron RJ et al, 2017 [80]	Inability to summarize the data
Wallace SS et Froum SJ, 2003 [4]	Inability to summarize the data
Yuen T, 2000 [81]	No free full text available
